# Safer Patients Empowered to Engage and Communicate about Health (SPEECH) in primary care: a feasibility study and process evaluation of an intervention for older people with multiple long-term conditions (multimorbidity)

**DOI:** 10.1186/s12875-023-02221-3

**Published:** 2024-01-05

**Authors:** Rebecca Goulding, Kelly Birtwell, Mark Hann, Sarah Peters, Harm van Marwijk, Peter Bower

**Affiliations:** 1 NIHR School for Primary Care Research, Centre for Primary Care and Health Services Research, School of Health Sciences, The University of Manchester, Manchester Academic Health Science Centre, Manchester, England; 2 Manchester Centre for Health Psychology, School of Health Sciences, The University of Manchester, Manchester Academic Health Science Centre, Manchester, England; 3 Department of Primary Care and Public Health, Brighton and Sussex Medical School, University of Brighton, Watson Building, Brighton, England

**Keywords:** Older adults, Multimorbidity, General practice, Primary care, Patient safety, Communication, Empowerment, Feasibility study.

## Abstract

**Background:**

Older people with multiple long-term conditions (multimorbidity) (MLTC-M) experience difficulties accessing and interacting with health and care services. Breakdowns in communication between patients and staff can threaten patient safety. To improve communication and reduce risks to patient safety in primary care, we developed an intervention: Safer Patients Empowered to Engage and Communicate about Health (SPEECH). SPEECH comprises a booklet for patients and an associated guide for staff. The booklet is designed to provide patients with information about staff and services, skills to prepare and explain, and confidence to speak up and ask.

**Methods:**

A single-arm mixed methods feasibility study with embedded process evaluation. General practices in the North West of England were recruited. Participating practices invited patients aged 65+ with MLTC-M who had an appointment scheduled during the study period. Patients were asked to complete questionnaires at baseline and follow-up (four to eight weeks after being sent the patient booklet), including the Consultation and Relational Empathy measure, Empowerment Scale, Multimorbidity Treatment Burden Questionnaire, and Primary Care Patient Measure of Safety. Staff completed questionnaires at the end of the study period. A sub-sample of patients and staff were interviewed about the study processes and intervention. Patients and the public were involved in all aspects of the study, from generation of the initial idea to interpretation of findings.

**Results:**

Our target of four general practices were recruited within 50 days of the study information being sent out. A fifth practice was recruited later to boost patient recruitment. We received expressions of interest from 55 patients (approx. 12% of those invited). Our target of 40 patient participants completed baseline questionnaires and were sent the SPEECH booklet. Of these, 38 (95%) completed follow-up. Patients found the intervention and study processes acceptable, and staff found the intervention acceptable and feasible to deliver.

**Conclusions:**

Our findings suggest the intervention is acceptable, and it would be feasible to deliver a trial to assess effectiveness. Prior to further evaluation, study processes and the intervention will be updated to incorporate suggestions from participants.

**Trial registration:**

The study was registered on the ISRCTN registry (ISRCTN13196605: 10.1186/ISRCTN13196605).

**Supplementary Information:**

The online version contains supplementary material available at 10.1186/s12875-023-02221-3.

## Background

Increasing numbers of people live with multiple long-term conditions (multimorbidity) (MLTC-M), including half of those aged 65+ [1]. These patients may be prescribed multiple medications, have unmet needs, and difficulties accessing and interacting with healthcare services [2, 3]. As such, older people with MLTC-M may be more likely to experience patient safety incidents.

Patient safety is defined as the “avoidance, prevention, and amelioration of adverse outcomes or injuries stemming from the processes of health care itself” (p.2) [4]. This encompasses issues from the wrong medication to a lack of trust in a provider. That is, both physical and psycho-social safety. The latter is a relational concept that “embraces the social elements of the interaction and their influence on people’s sense of identity as well as the purely psychological – what people think and feel” (p.256) [5].

Patient safety incidents have been found to occur in around 3% of primary care consultations [6]. Findings from the beginning of a longitudinal qualitative study suggest many patient safety incidents arise because of gaps in communication between patients and healthcare staff [7]. Communication issues can leave patients feeling dehumanised and disempowered, and threaten their psycho-social safety [8, 9]. In our earlier qualitative study patients reported avoiding contact with staff and said they “put off” making appointments when they experienced previous difficulties [7]. Communicating needs and experiences, and dealing with communication issues, is part of the healthcare process. However patients’ ability and capacity to do this will vary due to a range of factors including their health and health literacy [10, 11].

Care for older patients with MLTC-M can be provided most effectively and safely when there is relational continuity, as this enables patients to communicate openly, feel empowered and develop trust [12, 13]. However, such relational continuity is not always possible in primary care, and patients need to be able to communicate openly and feel safe with all healthcare staff.

Street et al. (2005) explored differences in how often patients express concerns and ask questions in consultations. These communication behaviours were found to be predominately patient-initiated, suggesting patient-orientated interventions could empower people to communicate more effectively, and improve patient safety [14]. A systematic review identified three such interventions for older people [15] with some positive results, but no definitive conclusions were possible and there continues to be a lack of high-quality research in this area. Furthermore, no such intervention has been developed for older people with MLTC-M, who could benefit most.

We sought to address this gap by drawing on relevant theories and carrying out primary research to identify how older people with MLTC-M (hereinafter referred to as patients) could be supported to communicate with primary care staff (papers in progress). Through a series of studies, Goulding [16] developed a brief behaviour change intervention entitled “SPEECH” (Safer Patients Empowered to Engage and Communicate about Health). The SPEECH intervention was designed and developed in collaboration with stakeholders (patients, carers, primary care staff and other experts). The present study assesses the feasibility of delivering and evaluating the SPEECH intervention.

### Aims and objectives

The aims of this study were to assess the usability and acceptability of the SPEECH intervention, and the feasibility of delivering this, in order to inform a subsequent effectiveness trial with older people with MLTC-M. Our specific objectives were to investigate:

A) Recruitment: the level of interest from general practices and patients, the time taken to recruit practices and the number of patients recruited.
B) Outcome measures: the number of questionnaires returned at baseline and follow-up, and the number of items completed per questionnaire.
C) Usability, acceptability, and feasibility: the usability and acceptability of the intervention, and the feasibility of delivering this in general practice.

## Methods

### Study design

This study was a single-arm mixed-methods feasibility study, in line with current guidance (www.rds-sw.nihr.ac.uk/dloads/RfPB_Feasibility_Trials_Guidance.pdf), with an embedded process evaluation. It was registered on the ISRCTN registry (ISRCTN13196605). The purpose of the process evaluation was to gain an understanding of the feasibility of the intervention, to further optimise its design, and to inform future evaluation [17]. We followed the CONSORT reporting guideline for testing the feasibility of several components of a trial [18]. The study was reviewed and approved by the London Bridge Research Ethics Committee (Ref: 20/LO/1284, 21/12/2020).

### Patient and Public Involvement (PPI)

The idea for this research arose from work with a PPI group on an earlier programme of research [19]. Empowerment was selected as a key outcome measure as this group emphasised the importance of the intervention supporting patients to feel more confident in healthcare interactions. Four public contributors met regularly throughout the current study. They helped with the development of non-standardised questionnaires and interview topic guides. The public contributors also supported recruitment by providing suggestions for and commenting on study materials. Following their input, the wording of study adverts and information sheets was revised to make these more understandable and appealing. Additionally, public contributors provided feedback on the research findings, informing our understanding of these, and plans for next steps.

We referred to guidelines on best practice [20] to ensure involvement was conducted to the highest standard, and we offered training and support for our public contributors.

### Participants and setting

General practices in the North West of England were invited to take part in the study. Fifteen practices were contacted directly by the research team due to their interest or involvement in earlier stages of the research. Further practices were contacted by an NIHR Local Clinical Research Network (LCRN). A Research Information Sheet for Practices (RISP) [21] was shared via email by the research team (December 2020 or January 2021) and the LCRN (December 2020). The RISP provided the following information: title and summary of the project, lead researcher, host institution, funder, details of ethical approval, type of data collection, what the researcher will do, what the practice staff will be asked to do, and information on consent and confidentiality. Interested practices were selected from a range of areas based on measures of relative deprivation [22]. Two were from areas with Index of Multiple Deprivation (IMD) deciles 1–3 and two were from areas with IMD deciles 4–6. The fifth practice was from an area with IMD decile 8. Recruitment of the fifth practice was based on the size of the population of patients aged 65+ (more than 2500 patients).

Patient participants were recruited between April and October 2021, through invitation letters sent by practice staff. Patients were eligible to take part if they (1) were aged 65 and over; (2) had two or more long-term conditions (at least two physical long-term conditions or a physical and mental health condition); (3) had capacity to provide informed consent; and (4) could read and write in English. Attempts were made to recruit patients with a general practice appointment during the study period, however this was a factor in slowing down recruitment at some sites. Clinical and administrative staff with direct patient contact were recruited from participating general practices. Interested potential patient and staff participants were asked to contact the research team. All participants provided written informed consent. Practices were reimbursed for staff time and patients were offered a shopping voucher.

A sample size calculation was deemed not necessary due to the aim of the study. Based on findings of a recent simulation study that recommended a minimum of 35 participants per group [23] we considered recruitment of 40 or more patient participants to the current single-arm feasibility study would provide preliminary evidence of feasibility of recruitment to a larger effectiveness trial. This number is also sufficient to estimate key parameters (e.g. the standard deviation of the potential primary outcome) to inform a power calculation for a future definitive trial with an adequate degree of precision [24]. Based on an estimate of 20% uptake, we anticipated 200 patients would need to be invited.

A sub-sample of participants (both patients and staff) were invited to participate in the process evaluation. Participants were sampled to represent the range of practices, with patient participants stratified according to self-reported use of the intervention, and staff participants by profession. We planned to recruit 16 patients and 8 staff members for the process evaluation from an estimated 32 staff members anticipated to complete questionnaire measures.

### Intervention

**Fig. 1:**
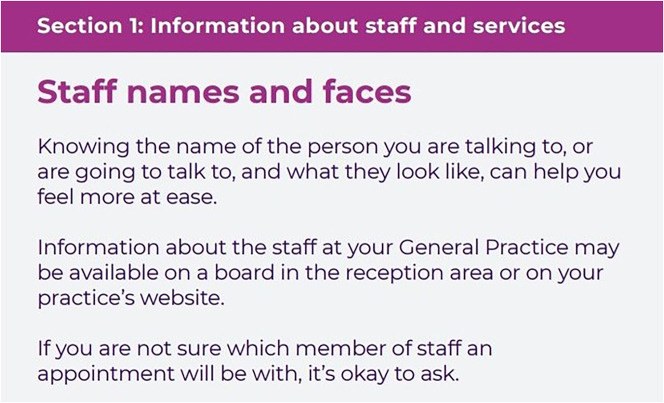
SPEECH patient booklet example extract, Section 1: Information about staff and services

**Fig. 2:**
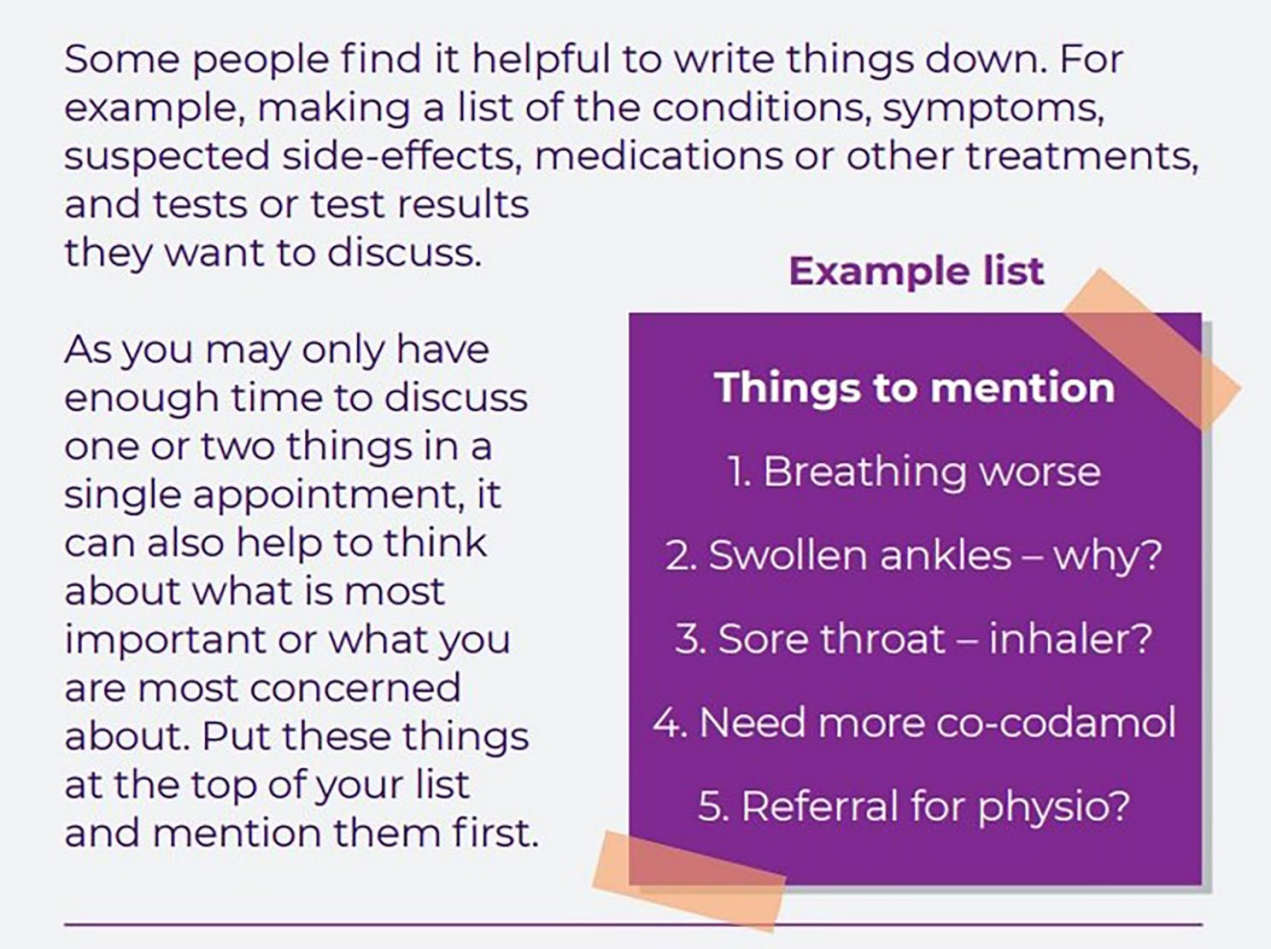
SPEECH patient booklet example extract, Section. 2: Skills to prepare and explain

**Fig. 3:**
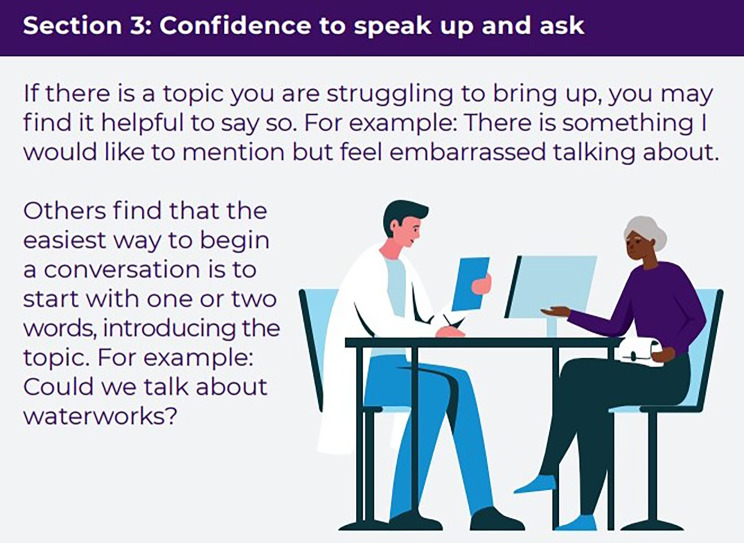
SPEECH patient booklet example extract, Section 3: Confidence to speak up and ask

The SPEECH intervention comprises an A5 booklet to be read and used by patients, and an A4 practice guide for staff explaining the need for and content of the patient booklet and other ways staff can support their patients to improve communication. The SPEECH intervention was developed using a theory-, evidence- and person-based approach [16]. Stakeholders (including patients and general practice staff) were involved throughout and helped to select the intervention content and suggest improvements to a prototype. The intervention incorporates six intervention functions and 11 behaviour change techniques [16, 25, 26]. The six intervention functions are: education, persuasion, training, modelling, enablement and environmental restructuring. The patient booklet contains three sections, to provide: (1) Information about staff and services; (2) Skills to prepare and explain; (3) Confidence to speak up and ask. A description of the intervention as per the TIDieR checklist [27] can be seen in Table 1. Example extracts from each section of the patient booklet can be seen in Figs. 1, 2 and 3. Patient participants were mailed a copy of the booklet, asked to read through it and follow the guidance within. Practice staff were asked to read both booklets.

**Table 1:** Description of the intervention as per the TIDieR checklist (Hoffmann et al. 2014)

No.	Item
**Brief name**	
1.	SPEECH (Safer Patients Empowered to Engage and Communicate about Health) – a patient-focused behaviour change intervention to empower older people (aged 65+) with multiple long-term conditions – multimorbidity (MLTC-M), improve their communication with staff working in general practices, and reduce risks to patient safety.
**Why**	
2.	A theory-, evidence- and person-based approach was used to plan, design and develop the intervention. Plan: Barriers to and enablers of communication were identified using the COM-B model of behaviour change. Design: Key stakeholders (including patients and staff) determined which barriers and enablers were the most important to address and promote, and discussed how this might be achieved. Alongside these discussions, the Behaviour Change Wheel was used to identify relevant intervention functions (mechanisms of action) and select Behaviour Change Techniques (active ingredients). Develop: The intervention was optimised through rounds of user feedback and modification. A logic model for the intervention is shown in Goulding [16].
**What**	
3.	Materials: Booklet for patients and guidance document for staff. The booklet for patients is the intervention. This has three main sections: (1) Information about staff and services, (2) Skills to prepare and explain, and (3) Confidence to speak up and ask. The guidance document for staff is designed to support the implementation of the intervention. This explains the purpose of the intervention and how practices and their staff can support the intervention.
4.	Procedures: Participating patients were given a copy of the intervention booklet. They were asked to read the booklet and make use of the suggestions and guidance within. If participating patients had any questions or queries about the booklet or its contents, they were able to contact the research team for support. The research team also contacted participating patients on one occasion after they received the booklet to ask if they needed support. Participating practices were given copies of both the intervention booklet and the implementation document. They were asked to circulate these materials to all staff who have contact with patients, and to make use of the suggestions and guidance within. If staff at participating practices had any questions or queries about the materials or their contents, they were able to contact the research team for support. The research team also contacted participating practices on one occasion after they received the materials to ask if they needed support.
**Who provided**	
5.	The research team provided the materials to participating patients and practices, and provided support as and when needed.
**How**	
6.	The materials were distributed by post and/or via email according to the participating patients’ and practices’ preferences. Support was provided by telephone, video-call or email according to the participating patients’ and practices’ preferences.
**Where**	
7.	NHS General Practices in North West England.
**When and how much**	
8.	Patients were given a copy of the intervention booklet at the start of their participation in the study, once they had provided informed consent and completed baseline questionnaires. After eight weeks, they were sent follow-up questionnaires and their participation in the study came to an end. Throughout the study period, participating patients were able to contact the research team for support. The research team also contacted participating patients two to four weeks after they received the booklet to ask if they needed support. Practices were given copies of the intervention booklet and implementation document at least two weeks before the first patient participant from their practice.
**Tailoring**	
9.	N/A
**How well**	
11.	At follow-up, eight weeks after being sent the intervention booklet, participating patients were asked to complete a pro forma to provide information on their engagement with the booklet, and whether or not they tried to make use of the suggestions and guidance within. At the end of the study period for all patient participants at a practice, staff were asked to complete a pro forma to provide information on their engagement with the intervention booklet and implementation document, and whether or not their practice tried to make use of the suggestions and guidance within the latter.

### Study assessments

Data were collected via questionnaires and interviews. At baseline, patient participants were asked to complete a demographic and background information form as well as the following measures: Empowerment Scale (ES) [28]; Consultation and Relational Empathy (CARE) measure [29]; Multimorbidity Treatment Burden Questionnaire (MTBQ) [30]; Primary Care Patient Measure of Safety (PC PMOS) [31]. The ES is a 47-item measure, designed to measure empowerment in primary care patients with long-term conditions. The CARE measure has 10 items and was developed as a patient-assessed measure of empathy in general practice settings, for patients from a range of socio-economic backgrounds. The MTBQ, a 10-item measure, was developed to assess treatment burden in older adults with MLTC-M in primary care. Global MTBQ scores can be grouped into four categories where 0 represents no burden, a score of under 10 represents low burden, 10–22 represents medium burden and over 22 represents high burden [30]. The PC PMOS is a 28-item measure to obtain patient feedback on the factors that contribute to patient safety in primary care. The PC PMOS advises patients to consider “interactions with your general practice during the last two months” when answering the questions. Aside from the categories for the MTBQ, we were unable to find reports of minimal clinically important differences (MCIDs) for these measures. Measures were mailed (by post or electronically) and completed independently, without a researcher present.

Four to eight weeks after being sent the SPEECH booklet, patient participants were asked to complete the same set of measures and a structured pro forma to provide information on their interaction with, use of, and views about the intervention (e.g. whether they would recommend the booklet to others – see additional file 1). This follow-up assessment point was to give a sense of change over time, retention rates for the feasibility study, and to give patients sufficient time to use the intervention booklet and provide feedback on its usability and acceptability. A longer assessment period would be used in a larger effectiveness trial.

Staff from participating practices completed assessments at only one timepoint: at the end of the implementation period (which lasted 4–5 months). Staff were asked to complete a demographic and background information form, and a structured pro forma with questions on their experience of and views on the delivery, usability and acceptability of the intervention (see additional file 2).

The sub-sample of participants in the process evaluation took part in semi-structured interviews at the end of the implementation period. During the interviews all participants were asked about their experiences of recruitment and data collection, and for their thoughts on the intervention. Interviews were conducted via telephone, were audio-recorded, and transcribed using intelligent verbatim transcription.

### Data analysis

We used descriptive statistics (with confidence intervals if appropriate) to assess the level of interest from general practices and patients, the time taken to recruit practices and the number of patients recruited.

We calculated the number of valid responses to each item of each questionnaire at baseline and follow-up. For the ES, CARE measure and PC PMOS, where applicable we excluded ‘don’t know’, ‘not applicable’, and missing items when calculating a total score on the questionnaire. For the MTBQ “does not apply” was scored zero, the same as “not difficult”, and missing items were excluded [30].

Total scores on each measure were calculated for each participant, assuming a sufficient number of items had been ‘completed’. For the ES, CARE measure and PC PMOS, the threshold was set to be at least 70% of items. This threshold was self-imposed as there was no specific guidance in the literature. For the MTBQ the threshold was 50%, as per the approach taken by the authors [30]. The participant-average item score was calculated across items that had been completed. This average item score was then multiplied by the number of items on the scale to ensure the total score reflects the true range of possible scores on the scale. This step is equivalent to assuming an ‘average response’ for missing items (including 'don’t know’ and ‘not applicable’ responses) for that participant. For the MTBQ, the average item score (possible range = 0–4) was multiplied by 25 to give a total score from 0 to 100 [30]. Scores on each outcome measure were descriptively summarised for both timepoints. Change scores (post-intervention minus pre-intervention) were also computed.

Interviews were transcribed and analysed using inductive qualitative content analysis [32] to explain and expand on the quantitative findings. Quotes are presented in the results, with pseudonyms.

## Results

### Recruitment: the level of interest from general practices and patients, the time taken to recruit practices and the number of patients recruited

Of the 15 practices contacted directly by the research team, five expressed an interest in participating. Of the practices contacted by the LCRN, nine expressed an interest in participating. From these, four general practices were recruited. The time taken to recruit, as measured by the time between the first RISP being sent out, and the agreement being signed, was 47 days. A further three practices expressed an interest but stated they did not have capacity at that time or would require support from a research nurse. After assessing patient recruitment part way through the study a fifth general practice was recruited, which took nine days. The Index of Multiple Deprivation (IMD) deciles, which were used to inform the selection of the participating practices were 2, 3, 6, 6 and 8, with 1 indicating the most deprived, and 10 indicating the least deprived areas [33].

In total, across the five practices, at least 466 invitations were sent to potential patient participants. We received expressions of interest from 55 patients (12%), with the proportion of expressions of interest ranging from 4 to 18% across the practices. Two patients (4%) were not eligible, and 8 (14%) could not be contacted. A further two (4%) expressed an interest in the study but later declined to take part due to health reasons.

**Fig. 4:**
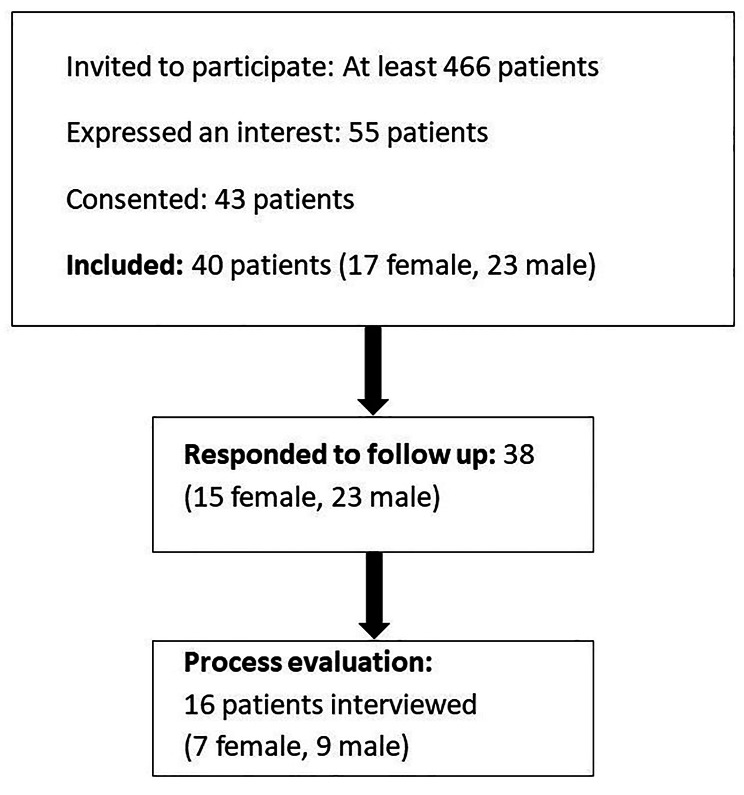
Patient participant recruitment

Although 43 patients completed consent forms for the study, due to problems with postal arrangements during the pandemic, two were received too late for inclusion. Additionally, one patient did not return the baseline questionnaires. As such, 40 patients (17 female, 23 male) participated (see Fig. 4). Further demographic data are presented in Table 2.

**Table 2:** Patient demographic data

Demographic characteristic		N	%
Gender	Female	17	42.5
	Male	23	57.5
Age	65–74	16	40.0
	75–84	20	50.0
	85+	4	10.0
Ethnicity	White British	40	100.0
Qualifications	No formal	10	25.0
	GCSE or equivalent	7	17.5
	A-Level or equivalent	6	15.0
	Degree	17	42.5
Living status	Alone	17	42.5
	With partner / children	23	57.5
Index of Multiple Deprivation (IMD) decile	1–3	6	15.0
	4–7	22	55.0
	8–10	12	30.0
Number of medications	< 5	15	37.5
	5–9	14	35.0
	10+	11	27.5
Number of self-reported long-term conditions	2–4	19	47.5
	5–6	11	27.5
	7+	10	25.0

Fifteen members of staff consented to take part and completed the study questionnaires. Demographic data for the staff participants are presented in Table 3.

**Table 3:** Staff demographic data

Demographic characteristic		N	%
Gender	Female	13	86.7
	Male	2	13.3
Age (N = 14)	35–44	4	28.6
	45–54	5	35.7
	55+	5	35.7
Ethnicity	White British	15	100.0
Professional role	GP	3	20.0
	Other clinical (e.g. nurse, Advanced Clinical Practitioner)	6	40.0
	Non-clinical (e.g. manager, administrator, receptionist)	6	40.0
Number of years in job/role type	1–4	5	33.3
	5–9	5	33.3
	10+	5	33.3

Sixteen patients (7 female, 9 male) and three primary care staff (all female) went on to participate in the process evaluation. Staff participants were from three different practices and patient participants were recruited from four different practices.

### Outcome measures: the number of questionnaires returned at baseline and follow-up, and the number of items completed per questionnaire

Questionnaire return rates were high: from the 41 patients whose completed consent forms were received in time, 40 sets of baseline questionnaires were returned (98%) and, of the 40 sent out, 38 sets of follow-up questionnaires were returned (95%). This indicates an attrition rate of 5% (confidence interval of 1–17%).

Completion rates for the individual questionnaires within each returned set varied. The Empowerment Scale (ES), Consultation and Relational Empathy (CARE) measure, and Multimorbidity Treatment Burden Questionnaire (MTBQ) were respectively completed by 95%, 95%, and 100% of participants at baseline and 95%, 97%, and 100% of participants at follow-up (see additional file 3). There were multiple items missing from the Primary Care Patient Measure of Safety (PC PMOS), with between 5 and 27 patients selecting “Not Applicable” as a response to each question. As such, the completion rates for this questionnaire were lower, with 70% completed at baseline and 61% at follow-up. Tables displaying the number of valid, missing, ‘not applicable’, and ‘don’t know’ responses for each standardised questionnaire item can be found in additional file 3. The follow-up proformas were completed by 36 (90%) participants. These proformas reported that 69% (25/36) had an appointment with their general practice since starting the study.

As reported in the development of the measure, there were relatively higher levels of “does not apply” selected for items 4, 6, 7, 9 and 10 of the MTBQ, compared to other MTBQ items [30]. These items were “monitoring medical conditions” (item 4), “seeing lots of different health professionals” (item 6), “attending appointments with health professionals” (item 7), “making recommended lifestyle changes” (item 9), and “having to rely on help from family and friends” (item 10). Levels of change from baseline to follow-up were calculated for all outcome measures, and can be found in additional file 3.

### Usability, acceptability, and feasibility: the usability and acceptability of the intervention, and the feasibility of delivering this in general practice

Usability and acceptability were assessed through proformas with questions about the materials and telephone interviews. The average duration of interviews was 19 min for patients (range: 9–36 min) and 33 min for staff (range: 24–42 min).

In response to questions on the patient proforma, 92% (33/36) stated the booklet was easy to understand, 86% (31/36) said they found it useful or that it will be useful in the future, and 81% (29/36) said they would recommend the booklet to others. Of the patient participants interviewed, all engaged with the patient booklet to some degree. The patient booklet was generally seen as usable and acceptable. Some patient participants thought no changes should be made as it would be hard to please everyone, and because the booklet was viewed favourably as it was: clear, simple, concise, engaging, and well set out. Some suggestions for change included: to use simpler language, make it shorter, change the order, broaden the remit (e.g. secondary as well as primary care, patients without MLTC-M), and offer alternative formats (e.g. braille or audio versions).

Three categories were generated from qualitative data on patient views of the booklet: Making a difference; Nothing new; In an ideal world.

1. Making a difference: The booklet was viewed positively, as something that suggested new ideas, gave confidence, or provided reassurance about things participants were already doing or had thought of doing. It was also seen as helping to improve the healthcare that participants received.

“it reminds you to just get your thoughts in order before you actually get on the phone. So, when you do speak to somebody, they’re able to actually give you either good advice or direct you to the right people very, very quickly.” (Philip).

“The booklet… it’s like my little shield now.” (Lesley).

2) Nothing new: Participants already did the things suggested in the booklet but thought it might be useful for others. However, when explored further, some participants admitted they were not undertaking the suggestions consistently and the booklet could be a useful reminder.

“It wasn’t particularly useful to me because most of the things it suggested, like I say, I already did, or were not appropriate. But I can see that it would be appropriate for other people with different problems to mine.” (Elizabeth).

3) In an ideal world: The booklet was viewed by some as idealistic, because it cannot address problems with access to healthcare professionals or perceived unhelpful attitudes of some staff members.

“this booklet gives the impression that all surgeries are the same, the staff are really nice helpful people who are falling over themselves to do what they can for you, and that’s not the case. So it’s really a bit idealistic, shall I say?” (Julie).

From the staff proforma, 93% (14/15) said they would use the materials again or in the future, and 93% said they would recommend the materials to patients and other practices. Findings of the staff interviews echoed those of patients in that the materials were seen as easy to understand and useful. However, some challenges with implementing the suggestions from the patient booklet were identified, e.g. how to address a list of issues brought by a patient when staff are short of time. Staff also discussed potential changes to the materials such as adding pages to the patient booklet so practice specific information could be added.

The study procedures were also discussed during interviews and all patients said they experienced no problems in taking part. It was suggested for a future study the interview questions could be provided in advance to help give participants time to think, and that conducting the interview over the phone helped put some participants at ease compared to being interviewed in-person.

All patients felt they benefitted from taking part in the research in some way, for example some patients benefitted from using the booklet through developing increased confidence. Some patients thought they were giving something back and helping to make a difference, and some patients found the research interesting and enjoyed taking part, being a part of something. This was seen as particularly helpful during the COVID-19 pandemic.

Staff members discussed the impact of the pandemic on their workload. It was thought this resulted in lower numbers of staff taking part in the study than originally anticipated, and staff could not engage as fully with the research. It was suggested that providing the staff leaflet in the form of a video or workshop with live examples of how to use the materials may encourage engagement. Other suggestions included bookmarks with reminders or prompts about the study, using an online survey format for questionnaires, and involving Primary Care Networks to support recruitment. It was also noted that pre-built searches for electronic records systems could help with identifying potential participants for a future trial.

In sum, these findings show the intervention was usable and acceptable to patients and staff, and the research was feasible to deliver. Practices successfully identified and invited patients to participate despite challenges and increased workload due to the COVID-19 pandemic, and patients experienced no problems taking part and felt they benefited from participation.

## Discussion

Findings of this feasibility study indicate that our brief behaviour change intervention to improve communication between older people aged 65+, with MLTC-M and primary care staff is acceptable, usable, and useful for patients and primary care staff. There was a good level of interest from general practices and we successfully recruited general practices and patient participants, although rates of expressions of interest from patients were below initial estimates. The study had a very low attrition rate, with 95% of participants returning the follow-up questionnaires. We assessed several uncertainties, meeting criteria for progression to a full randomised controlled trial. However, alternative study designs could be considered. For example, a theory-based realist approach could be used to explore how the intervention works, for whom, and in what circumstances [33].

We considered recruitment of 40 or more patient participants to the current feasibility study would provide preliminary evidence of feasibility of recruitment to a larger effectiveness trial. We met this target, recruiting 40 patient participants (although this represented 9% of the invited patients, rather than the 20% estimated). As noted above, we received further completed consent forms but were unable to include these patients in the study. Any follow up study should plan to send more invitation letters (our results suggest that in order to recruit 400 patients to a larger trial, approximately 4,700 study invitation letters would need to be sent) but also increase the proportion of those responding (to reduce bias in the sample).

This study was conducted during the COVID-19 pandemic which affected recruitment and engagement in a range of ways. For example, staff engagement was limited due to increased workloads. Due to limited opportunities for social activities and some people still shielding, it may be that some patient participants had more time to take part in the study, which may not be the case in a future trial.

The results would support the use of the Empowerment Scale as the primary outcome for a future trial as it performed well in terms of completion. This also aligns with input from our public contributors, and with the aim of the intervention. However, some participants reported they already did some of the things suggested in the SPEECH booklet, which may reflect the tension in research that people who are already somewhat empowered may be more likely to participate in studies such as this. The CARE measure and MTBQ were also well completed. The CARE measure has fewer items which may be more appealing to some participants.

The PC PMOS was poorly completed with “Not Applicable” selected in response to many questions. There could be several reasons for this. The guidance states: “Please read each statement carefully, keeping in mind your interactions with your general practice during the last two months, and circle one option for each question. If you have had no experience of a statement, please circle N/A, “Not Applicable”.” According to the follow-up proforma results, just under one third of patients had not had a general practice appointment during the study period. This might explain the high level of “Not Applicable” responses. There might also have been different interpretations of the guidance. ‘Interactions’ could have been interpreted to mean appointments rather than any telephone call or conversation, and ‘general practice’ as meaning their general practitioner or clinical staff only, rather than any member of staff at their practice. Participants may also have thought interactions should be face-to-face only (rather than via other means), which were less frequent at the time the data were being collected [34]. This was reflected in some of the informal conversations between study participants and members of the research team when we telephoned participants to check the study materials had arrived. The PC PMOS has been validated and used in populations with a mean age of 44–56 [35–37] whereas our participants were all aged over 65. Additional guidance and clarification may be required to support older populations to accurately complete the PC PMOS, and a wider timeframe for general practice interactions may be needed for some research studies.

Patient and Public Involvement was a strength of the current study. Members of the PPI group advised on study materials and outcome measures, which may have contributed to the very low attrition rate. In addition, the design and development of the intervention was informed by the needs and views of stakeholders including patients with MLTC-M [16]. This may have contributed to the acceptability and usability of the intervention.

Recruitment across several general practices was a further strength of the study. There was a good level of interest and practices were recruited within sufficient time to complete the study. However, only two of the participating practices were from deprived areas as ranked by the IMD. In addition, some practices expressed an interest in taking part but did not have capacity. Recruitment support for practices should be factored into a future trial, particularly for practices in deprived areas.

The main limitation of this study is the lack of diversity in the patient participant sample. All participants were white British, almost half were educated to degree level, and only 15% of participants were from the most deprived areas as ranked by the IMD. Potential participants were initially contacted by practice staff based on the study eligibility criteria, and from there our sample was self-selected. We did not explore barriers and reasons for not participating. Recruitment took place during the second year of the COVID-19 pandemic when there were fewer routine face-to-face appointments and the research team were not able to engage in face-to-face promotion of the study. A wider range of recruitment methods and greater community engagement could be employed in a future trial, and recent recommendations on how to improve inclusion in clinical trials may also support recruitment and retention of a more diverse range of participants [38]. This is needed in order to understand potential acceptability issues, which may be particularly important in older populations as the roles of family members may differ due to cultural factors. In addition, while recruitment of 40 participants provides preliminary evidence of feasibility of recruitment to a larger effectiveness trial, this is only one factor and the success of a future trial cannot be guaranteed based on this factor alone.

## Conclusions

Our brief behaviour change intervention to improve communication between patients and primary care staff is acceptable, usable, and useful for patients and primary care staff. The findings suggest a larger study would be feasible, and for a randomised controlled trial the Empowerment Scale may be a suitable primary outcome. Other options include conducting a theory-based realist evaluation. Patients and staff made important suggestions for changes to both the intervention and the research, in order to increase acceptability and engagement. Participants also suggested the intervention could be useful for other patient populations (e.g. secondary care patients and patients without MLTC-M), however this would require further development and testing. This intervention has the potential to empower patients, improve communication between patients and staff, and reduce risks to patient safety. We will continue to work with key stakeholders, including patients, carers, staff and other experts, to further refine and evaluate the intervention.

### Electronic supplementary material

Below is the link to the electronic supplementary material.

**Additional file 1:** SPEECH feasibility study patient proforma V2 11/07/2021. Proforma questionnaire given to patient participants at follow-up.

**Additional file 2:** SPEECH feasibility study staff proforma V2 11/07/2021. Proforma questionnaire given to staff participants at the end of the implementation period.

**Additional file 3:** Tables of questionnaire item responses and questionnaire levels of change information. Tables showing the number of valid, ‘not applicable’, ‘don’t know’, and missing responses for each questionnaire item, and levels of change for each questionnaire.

## Data Availability

The datasets generated and analysed during the current study are not publicly available due to the scope of consent provided by participants. As such, the datasets are available from the corresponding author, to other researchers from The University of Manchester, on reasonable request.

## Abbreviations

CARE measure: Consultation and Relational Empathy measure
ES: Empowerment Scale
GP: General Practitioner
IMD: Index of Multiple Deprivation
LCRN: Local Clinical Research Network
MLTC-M: Multiple Long-Term Conditions (Multimorbidity)
MTBQ: Multimorbidity Treatment Burden Questionnaire
PC PMOS: Primary Care Patient Measure of Safety
PPI: Patient and Public Involvement
RISP: Research Information Sheet for Practices
SPEECH: Safer Patients Empowered to Engage and Communicate about Health
TIDieR checklist: Template for Intervention Description and Replication (TIDieR) checklist and guide
