# Supplementary material for: Safer Patients Empowered to Engage and Communicate about Health (SPEECH) in primary care: a feasibility study and process evaluation of an intervention for older people with multiple long-term conditions (multimorbidity)
Source: BMC Prim Care. 2024 Jan 5;25:12. doi: 10.1186/s12875-023-02221-3 (PMC10768368; doi:10.1186/s12875-023-02221-3)
Supplement: Supplementary file 1 — Additional file 1: SPEECH feasibility study patient proforma V2 11/07/2021. Proforma questionnaire given to patient participants at follow-up. [file 12875_2023_2221_MOESM1_ESM.docx]

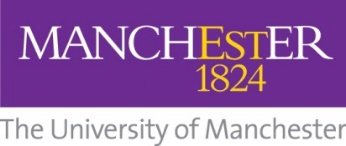

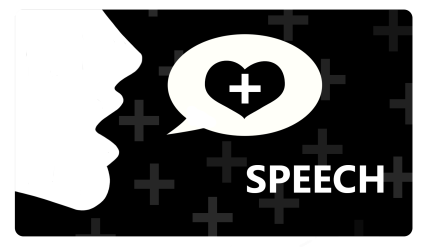


**Safer Patients Empowered to Engage and Communicate about Health (SPEECH) in primary care: a feasibility study and process evaluation of an intervention for older people with multiple long-term conditions (multimorbidity)**

Rebecca Goulding^*, Kelly Birtwell^1^^, Mark Hann, Sarah Peters, Harm van Marwijk, Peter Bower.

^ Joint first authors

*Corresponding authors: Rebecca.goulding@manchester.ac.uk; Kelly.birtwell@manchester.ac.uk

**Improving patient safety and communication**

Participant ID _______________

| **PATIENT PROFORMA** |
| --- |

The following questions ask you about your view on and use of the materials we provided to you at the beginning of the study

1. Did you read the booklet ‘How to get the most out of your General Practice’?

Not at all Once A few times Many times

2. I found the booklet easy to understand

Strongly agree Agree No opinion Disagree Strongly disagree

3. I think the booklet is / will be useful

Strongly agree Agree No opinion Disagree Strongly disagree

3a. I found section 1 of the booklet on 'Information about staff and services’ useful

Strongly agree Agree No opinion Disagree Strongly disagree

3b. I found section 2 of the booklet on ‘Skills to prepare and explain’ useful

Strongly agree Agree No opinion Disagree Strongly disagree

3c. I found section 3 of the booklet on ‘Confidence to speak up and ask’ useful

Strongly agree Agree No opinion Disagree Strongly disagree

4. Have you used any of the information and suggestions from the booklet?

Not at all Once A few times Many times

5. I would use the booklet again or in the future

Strongly agree Agree No opinion Disagree Strongly disagree

6. I would recommend the booklet to other people

Strongly agree Agree No Opinion Disagree Strongly disagree

7. How many appointments (in-person or via telephone or video-call) have you had with someone from your General Practice since you started the study?

………………

7a. What type(s) of appointment have you had? (please tick all that apply)

Appointment for a new problem with the GP

Follow-up appointment with the GP

Appointment with the nurse

Other appointment

8. How many other contacts (for example, phone calls to make an appointment or request a repeat prescription) have you had with someone from your General Practice since you started the study?

………………

9. If you have any other comments about the booklet, please write them below:

…………………………………………………………………………………………………………………………………

…………………………………………………………………………………………………………………………………

…………………………………………………………………………………………………………………………………

…………………………………………………………………………………………………………………………………

…………………………………………………………………………………………………………………………………
