# Supplementary material for: Safer Patients Empowered to Engage and Communicate about Health (SPEECH) in primary care: a feasibility study and process evaluation of an intervention for older people with multiple long-term conditions (multimorbidity)
Source: BMC Prim Care. 2024 Jan 5;25:12. doi: 10.1186/s12875-023-02221-3 (PMC10768368; doi:10.1186/s12875-023-02221-3)
Supplement: Supplementary file 2 — Additional file 2: SPEECH feasibility study staff proforma V2 11/07/2021. Proforma questionnaire given to staff participants at the end of the implementation period. [file 12875_2023_2221_MOESM2_ESM.docx]

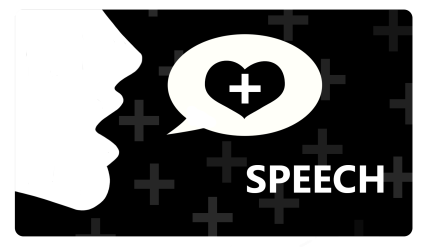

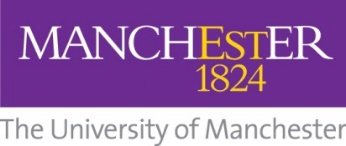
**Safer Patients Empowered to Engage and Communicate about Health (SPEECH) in primary care: a feasibility study and process evaluation of an intervention for older people with multiple long-term conditions (multimorbidity)**

Rebecca Goulding^*, Kelly Birtwell^1^^, Mark Hann, Sarah Peters, Harm van Marwijk, Peter Bower.

^ Joint first authors

*Corresponding authors: Rebecca.goulding@manchester.ac.uk; Kelly.birtwell@manchester.ac.uk

**Improving patient safety and communication**

Participant ID _______________

| **STAFF PROFORMA** |
| --- |

The following questions ask you about your views on and experience of the materials we provided to your practice and patients at your practice at the beginning of the study

1. Did you read the ‘Practice guide to SPEECH and the patient booklet’?

Not at all Once A few times Many times

2. Did you read the patient booklet ‘How to get the most out of your General Practice’?

Not at all Once A few times Many times

3. Did the practice discuss the materials as a team?

Not at all Once A few times Many times

4. I thought the suggestions on page 4 of the practice guide would be useful to patients

Strongly agree Agree No opinion Disagree Strongly disagree

5. I thought the content of the patient booklet would be useful to patients

Strongly agree Agree No opinion Disagree Strongly disagree

6. Have you or the practice used the materials?

Not at all Once A few times Many times

7. I would use the materials again or in the future

Strongly agree Agree No opinion Disagree Strongly disagree

8. I would recommend the booklet to patients

Strongly agree Agree No opinion Disagree Strongly disagree

9. I would recommend the materials to other practices

Strongly agree Agree No opinion Disagree Strongly disagree

10. How many times have you been in contact with patients in the study?

………………

11. I noticed a change in how patients in the study communicated with me or the practice

Strongly agree Agree No opinion Disagree Strongly disagree

12. If you have any other comments about the materials or their use by patients or the practice, please write them below:

…………………………………………………………………………………………………………………………………

…………………………………………………………………………………………………………………………………

…………………………………………………………………………………………………………………………………

…………………………………………………………………………………………………………………………………

…………………………………………………………………………………………………………………………………
