# Supplementary material for: Safer Patients Empowered to Engage and Communicate about Health (SPEECH) in primary care: a feasibility study and process evaluation of an intervention for older people with multiple long-term conditions (multimorbidity)
Source: BMC Prim Care. 2024 Jan 5;25:12. doi: 10.1186/s12875-023-02221-3 (PMC10768368; doi:10.1186/s12875-023-02221-3)
Supplement: Supplementary file 3 — Additional file 3: Tables of questionnaire item responses and questionnaire levels of change information. Tables showing the number of valid, ‘not applicable’, ‘don’t know’, and missing responses for each questionnaire item, and levels of change for each questionnaire. [file 12875_2023_2221_MOESM3_ESM.docx]

**Safer Patients Empowered to Engage and Communicate about Health (SPEECH) in primary care: a feasibility study and process evaluation of an intervention for older people with multiple long-term conditions (multimorbidity)**

Rebecca Goulding^*, Kelly Birtwell^1^^, Mark Hann, Sarah Peters, Harm van Marwijk, Peter Bower.

^ Joint first authors

*Corresponding authors: Rebecca.goulding@manchester.ac.uk; Kelly.birtwell@manchester.ac.uk

**Tables of questionnaire item responses and questionnaire levels of change information**

Table 1: Number of valid and missing responses – ES Questionnaire

Table 2: Number of valid, not applicable and missing responses – CARE Questionnaire

Table 3: Number of valid, not applicable and missing responses – MTBQ Questionnaire

Table 4: Number of valid, not applicable, don’t know, and missing responses – PC PMOS Questionnaire

Table 5: Levels of change for all four outcome measures

Figure 1: Level of change for the Empowerment Scale

**Table 1: Number of valid and missing responses – ES Questionnaire**

|  | Baseline | | Follow-Up | |
| --- | --- | --- | --- | --- |
|  | Valid | Missing | Valid | Missing |
| 1 | 40 | 0 | 38 | 2 |
| 2 | 40 | 0 | 38 | 2 |
| 3 | 39 | 1 | 36 | 4 |
| 4 | 40 | 0 | 38 | 2 |
| 5 | 40 | 0 | 38 | 2 |
| 6 | 40 | 0 | 38 | 2 |
| 7 | 40 | 0 | 37 | 3 |
| 8 | 40 | 0 | 38 | 2 |
| 9 | 39 | 1 | 37 | 3 |
| 10 | 39 | 1 | 37 | 3 |
| 11 | 39 | 1 | 37 | 3 |
| 12 | 38 | 2 | 37 | 3 |
| 13 | 38 | 2 | 37 | 3 |
| 14 | 37 | 3 | 37 | 3 |
| 15 | 38 | 2 | 35 | 5 |
| 16 | 38 | 2 | 37 | 3 |
| 17 | 39 | 1 | 37 | 3 |
| 18 | 38 | 2 | 37 | 3 |
| 19 | 39 | 1 | 37 | 3 |
| 20 | 38 | 2 | 37 | 3 |
| 21 | 39 | 1 | 36 | 4 |
| 22 | 38 | 2 | 37 | 3 |
| 23 | 39 | 1 | 37 | 3 |
| 24 | 39 | 1 | 35 | 5 |
| 25 | 39 | 1 | 35 | 5 |
| 26 | 38 | 2 | 34 | 6 |
| 27 | 39 | 1 | 34 | 6 |
| 28 | 39 | 1 | 35 | 5 |
| 29 | 38 | 2 | 35 | 5 |
| 30 | 39 | 1 | 35 | 5 |
| 31 | 39 | 1 | 35 | 5 |
| 32 | 38 | 2 | 35 | 5 |
| 33 | 37 | 3 | 34 | 6 |
| 34 | 38 | 2 | 35 | 5 |
| 35 | 37 | 3 | 35 | 5 |
| 36 | 37 | 3 | 35 | 5 |
| 37 | 39 | 1 | 35 | 5 |
| 38 | 38 | 2 | 38 | 2 |
| 39 | 39 | 1 | 38 | 2 |
| 40 | 39 | 1 | 37 | 3 |
| 41 | 39 | 1 | 37 | 3 |
| 42 | 39 | 1 | 36 | 4 |
| 43 | 39 | 1 | 37 | 3 |
| 44 | 38 | 2 | 37 | 3 |
| 45 | 38 | 2 | 36 | 4 |
| 46 | 39 | 1 | 37 | 3 |
| 47 | 39 | 1 | 38 | 2 |

**Table 2: Number of valid, not applicable and missing responses – CARE Questionnaire**

|  | Baseline | | | Follow-Up | | |
| --- | --- | --- | --- | --- | --- | --- |
|  | Valid | N/A | Missing | Valid | N/A | Missing |
| 1 | 39 | 1 | 0 | 37 | 0 | 3 |
| 2 | 39 | 1 | 0 | 36 | 1 | 3 |
| 3 | 39 | 1 | 0 | 35 | 2 | 3 |
| 4 | 38 | 2 | 0 | 37 | 0 | 3 |
| 5 | 39 | 1 | 0 | 37 | 0 | 3 |
| 6 | 38 | 1 | 1 | 37 | 0 | 3 |
| 7 | 39 | 0 | 1 | 37 | 0 | 3 |
| 8 | 38 | 1 | 1 | 37 | 0 | 3 |
| 9 | 36 | 3 | 1 | 35 | 2 | 3 |
| 10 | 36 | 3 | 1 | 36 | 1 | 3 |

**Table 3: Number of valid, not applicable and missing responses – MTBQ Questionnaire**

|  | Baseline | | | Follow-Up | | |
| --- | --- | --- | --- | --- | --- | --- |
|  | Valid | N/A | Missing | Valid | N/A | Missing |
| 1 | 35 | 5 | 0 | 36 | 2 | 2 |
| 2 | 40 | 0 | 0 | 38 | 0 | 2 |
| 3 | 37 | 3 | 0 | 36 | 2 | 2 |
| 4 | 29 | 10 | 1 | 28 | 10 | 2 |
| 5 | 40 | 0 | 0 | 38 | 0 | 2 |
| 6 | 34 | 5 | 1 | 32 | 6 | 2 |
| 7 | 34 | 6 | 0 | 33 | 5 | 2 |
| 8 | 39 | 1 | 0 | 37 | 1 | 2 |
| 9 | 36 | 4 | 0 | 32 | 6 | 2 |
| 10 | 33 | 7 | 0 | 31 | 7 | 2 |

**Table 4: Number of valid, not applicable, don’t know, and missing responses – PC PMOS Questionnaire**

|  | Baseline | | | | Follow-Up | | | |
| --- | --- | --- | --- | --- | --- | --- | --- | --- |
|  | Valid | N/A | D/K | Missing | Valid | N/A | D/K | Missing |
| 1 | 31 | 9 | 0 | 0 | 28 | 8 | 0 | 4 |
| 2a | 26 | 13 | 1 | 0 | 20 | 15 | 0 | 5 |
| 2b | 27 | 12 | 0 | 1 | 22 | 13 | 0 | 5 |
| 2c | 24 | 14 | 2 | 0 | 15 | 19 | 0 | 6 |
| 2d | 20 | 20 | 0 | 0 | 17 | 17 | 0 | 6 |
| 3 | 29 | 10 | 1 | 0 | 22 | 13 | 0 | 5 |
| 4 | 29 | 10 | 0 | 1 | 24 | 11 | 0 | 5 |
| 5 | 27 | 12 | 0 | 1 | 27 | 9 | 0 | 4 |
| 6 | 29 | 10 | 0 | 1 | 25 | 10 | 0 | 5 |
| 7 | 27 | 10 | 2 | 1 | 20 | 14 | 1 | 5 |
| 8 | 28 | 9 | 2 | 1 | 24 | 10 | 1 | 5 |
| 9 | 16 | 23 | 0 | 1 | 11 | 24 | 0 | 5 |
| 10 | 10 | 26 | 3 | 1 | 7 | 27 | 1 | 5 |
| 11 | 30 | 9 | 0 | 1 | 25 | 10 | 0 | 5 |
| 12 | 30 | 9 | 0 | 1 | 22 | 12 | 0 | 6 |
| 13 | 26 | 9 | 4 | 1 | 20 | 13 | 2 | 5 |
| 14 | 17 | 22 | 0 | 1 | 16 | 19 | 0 | 5 |
| 15 | 28 | 11 | 0 | 1 | 24 | 11 | 0 | 5 |
| 16 | 30 | 7 | 1 | 2 | 26 | 9 | 0 | 5 |
| 17 | 15 | 24 | 0 | 1 | 17 | 18 | 0 | 5 |
| 18 | 30 | 9 | 0 | 1 | 29 | 6 | 0 | 5 |
| 19 | 28 | 9 | 1 | 2 | 25 | 9 | 0 | 6 |
| 20 | 32 | 7 | 0 | 1 | 26 | 8 | 0 | 6 |
| 21 | 31 | 7 | 0 | 2 | 26 | 8 | 0 | 6 |
| 22 | 33 | 5 | 0 | 2 | 29 | 5 | 0 | 6 |
| 23 | 32 | 6 | 0 | 2 | 26 | 8 | 0 | 6 |
| 24 | 30 | 7 | 1 | 2 | 26 | 7 | 1 | 6 |
| 25 | 32 | 7 | 0 | 1 | 27 | 6 | 0 | 7 |

**Levels of change:** The Empowerment Scale showed the greatest average absolute level of change between baseline and follow-up (+8.2 points). Approximately 75% of patients felt more empowered (to various degrees). On average, change in scores from baseline were much more modest on the other three outcomes. Levels of change for all four outcome measures can be seen in table 5, level of change for the Empowerment Scale can be seen in figure 1.

**Table 5: Levels of change for all four outcome measures**

|  | Baseline | Follow-Up | Change  (Follow-Up – Baseline) |
| --- | --- | --- | --- |
| Empowerment Scale |  |  |  |
| possible data range | 47 – 235 | 47 – 235 | -188 – 188 |
| N (completed ≥33/47 items) | 38 | 36 | 35 |
| mean (SD) | 162.4 (18.7) | 171.1 (19.9) | 8.2 (14.0) |
| median (IQR) | 159.5 (151, 179) | 175.1 (165.8, 183.5) | 7 (-3, 14.7) |
| observed data range | 114.4 – 200.3 | 120 – 203 | -12.2 – 45 |
|  |  |  |  |
| CARE |  |  |  |
| possible data range | 10 – 50 | 10 – 50 | -40 – 40 |
| N (completed ≥7/10 items) | 38 | 37 | 36 |
| mean (SD) | 33.3 (11.2) | 33.0 (11.2) | -1.3 (9.0) |
| median (IQR) | 34 (25, 41) | 35 (24, 40) | -1 (-6, 2.5) |
| observed data range | 11 – 50 | 10 – 50 | -24 – 26 |
|  |  |  |  |
| MTBQ |  |  |  |
| possible data range | 0 – 100 | 0 – 100 | -100 – 100 |
| N (completed ≥5/10 items) | 40 | 38 | 38 |
| mean (SD) | 16.5 (14.8) | 14.2 (11.7) | -2.0 (8.7) |
| median (IQR) | 13.75 (5, 22.5) | 10 (5, 25) | 0 (-7.5, 2.5) |
| observed data range | 0 – 62.5 | 0 – 45 | -17.5 – 22.5 |
|  |  |  |  |
| PC PMOS |  |  |  |
| possible data range | 28 – 140 | 28 – 140 | -112 – 112 |
| N (completed ≥20/28 items) | 28 | 23 | 20 |
| mean (SD) | 105.6 (15.7) | 104.1 (17.0) | -0.1 (7.7) |
| median (IQR) | 106.7 (95.8, 112.6) | 105 (93.3, 114.8) | 0.9 (-2.0, 3.6) |
| observed data range | 68.2 – 137.8 | 75.5 – 140 | -21.6 – 9.8 |

**Figure 1: Level of change for the Empowerment Scale**
